# Surgical Surprise: A Rare Chest Wall Muscle during Gynecomastia Surgery

**DOI:** 10.1055/s-0045-1802951

**Published:** 2025-02-10

**Authors:** Joyal Jose, Deepak Aravind

**Affiliations:** 1 Department of Plastic & Reconstructive Surgery, Believers Church Medical College Hospital, Thiruvalla, Kerala, India

Sir,

Gynecomastia correction is one of the commonest aesthetic surgeries performed by a plastic surgeon. The sternalis muscle is a rare chest wall muscle, usually described in the literature by anatomists, during cadaveric dissections. 1 There are only a few clinical case reports regarding the same in aesthetic breast surgeries. 2 The position of the muscle adjacent to the medial border of breast tissue makes it significant in all types of breast surgeries. We describe our clinical experience of encountering this muscle during gynecomastia surgery.

**Fig. 1:**
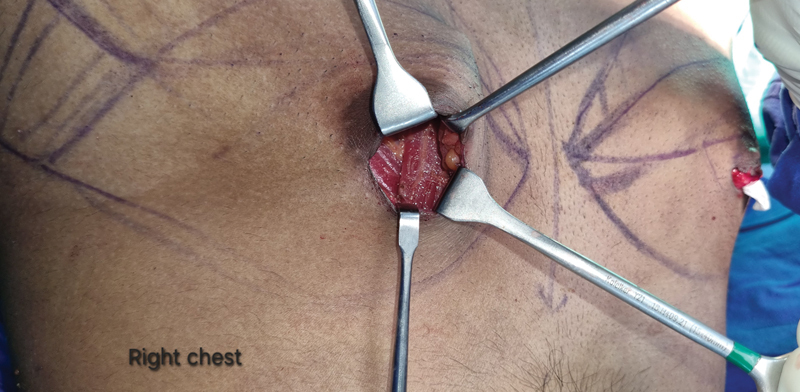
Vertical muscle fibers: right parasternal region, anterior to the pectoralis major muscle fibers.

**Fig. 2:**
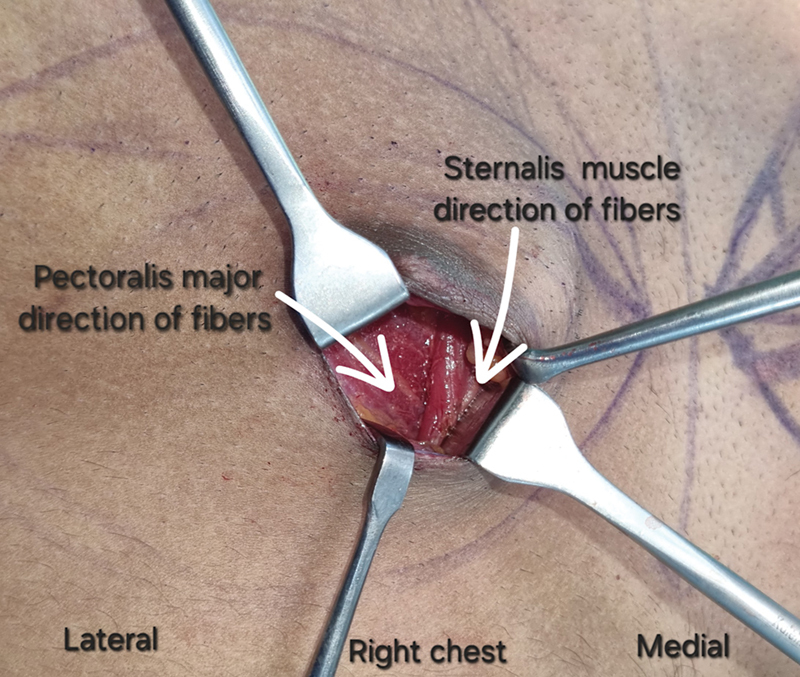
Orientation of muscle fibers of the sternalis and pectoralis major ( *white arrows* ).

A 29-year-old male bodybuilder with bilateral Simon grade 2a gynecomastia was planned for chest liposuction followed by gland excision. After liposuction, gland excision proceeded with the inferior periareolar incision. During right-side gland excision, a parasternal vertical bulge of muscle was noted, anterior to the pectoralis major ( Figs. 1 and 2 ). The orientation of those muscle fibers was perpendicular to the pectoralis major and it was found to be a separate muscle. The same finding was noted on the opposite side, though smaller in size. These extra muscle fibers were left alone and gland excision was completed. Postoperatively it was explained to the patient. We did some literature search for the same, and from previous case reports and cadaveric study data, it was found to be a muscle named “STERNALIS.” A mild medial chest prominence was noted postoperatively, which can be attributed to the hypertrophied sternalis muscles in this patient.

First described in detail by Dupuy (1726), the incidence of this muscle in cadaveric studies varies depending on the region and race. In the Indian population, its incidence is 4 to 8% in both genders. 3 The origin of the muscle is variable and is postulated to arise from adjacent muscles like the sternocleidomastoid/rectus abdominis/pectoralis major. The sternalis muscle is described to be innervated from external/internal thoracic nerves/intercostal nerves. 4 Is significance in plastic surgery, especially breast surgery, is multifold like interference with mammography interpretation, 5 interference with internal mammary artery (IMA) pedicle dissection in breast reconstruction, and interference with submuscular pocket dissection in augmentation mammoplasty. 5 It can coexist with pectoralis major defects. 4

The sternalis muscle, a rare chest wall muscle, is known more by anatomists than surgeons. The clinical presence of this muscle should be identified and reported. More light has to be shed on the vascular anatomy and innervation of the muscle for its clinical use. The clinical significance of this muscle in relation to aesthetic breast surgeries should be studied.
